# Epithelioid Angiosarcoma Masquerading as Multiple Myeloma: A Diagnostic Pitfall

**DOI:** 10.7759/cureus.119129

**Published:** 2026-11-04

**Authors:** John Fahy, Gayatri Dhar, Lauren Kandakumar, Shinnosuke Kitaoka, Ei Ei Phyu

**Affiliations:** 1 General Medicine, Mid and South Essex NHS Foundation Trust, Southend, GBR; 2 Radiology, Mid and South Essex NHS Foundation Trust, Southend, GBR; 3 Geriatric Medicine, Mid and South Essex NHS Foundation Trust, Southend, GBR

**Keywords:** bone tumour, cytokeratin, diagnostic pitfall, epithelioid angiosarcoma, lytic bone lesions, multiple myeloma, plasma-cell neoplasm

## Abstract

Multifocal osteolytic lesions in an older adult, particularly in the presence of hypercalcaemia and a monoclonal paraprotein, raise strong suspicion of multiple myeloma. However, coexisting pathological processes may produce a convincing but misleading clinical picture.

We describe a case involving a man in his 80s who presented with back pain, hypercalcaemia and multifocal lytic vertebral lesions, together with an elevated kappa/lambda free light-chain ratio and a monoclonal serum band. Multiple myeloma was initially considered the leading diagnosis. Bone marrow aspirate and trephine biopsy, however, demonstrated only a low-volume kappa-restricted plasma-cell neoplasm, with approximately 3%-4% plasma cells. Further examination identified small foci of cytokeratin-positive cohesive cells, initially raising concern for metastatic carcinoma.

The discrepancy between the extensive radiological disease burden and limited plasma-cell findings prompted targeted biopsy of an L3 lytic lesion. This demonstrated a separate population of atypical epithelioid cells expressing pancytokeratin (AE1/3), ERG and CD31, which supported vascular differentiation despite aberrant cytokeratin expression. Following a multidisciplinary review of the clinical, radiological and histopathological findings, a likely diagnosis of epithelioid angiosarcoma was made. A low-volume kappa-restricted plasma-cell neoplasm was again identified in the background.

The patient's condition deteriorated rapidly, and he died before the final diagnosis was confirmed. This case demonstrates how epithelioid angiosarcoma can masquerade as multiple myeloma, particularly when a coexisting plasma-cell neoplasm provides a plausible but incomplete explanation for extensive lytic bone disease. Discordance between radiological disease burden and pathological findings should prompt reconsideration of the working diagnosis and timely targeted biopsy of radiologically abnormal tissue.

## Introduction

Multifocal osteolytic lesions in an older adult, with hypercalcaemia and a monoclonal paraprotein, raise strong suspicion of multiple myeloma. Nevertheless, the presence of a monoclonal protein alone does not confirm the diagnosis. Current diagnostic criteria require evidence of a clonal plasma-cell disorder together with myeloma-defining events; thus, careful clinicopathological correlation remains essential when the extent of plasma-cell involvement does not reflect the radiological findings [1].

Primary vascular neoplasms of bone are rare, with angiosarcoma accounting for less than 1% of malignant bone tumours [2]. Multicentric osseous angiosarcoma may present with multifocal osteolytic disease and has previously been reported to mimic multiple myeloma [3]. Epithelioid angiosarcoma presents an additional diagnostic challenge, as it can express epithelial markers, including cytokeratins, alongside endothelial markers such as CD31 (platelet endothelial cell adhesion molecule-1) and ERG (ETS-related gene), potentially resulting in confusion with metastatic carcinoma [4].

We present a case of epithelioid angiosarcoma involving bone that initially appeared highly suggestive of multiple myeloma because of concurrent hypercalcaemia, monoclonal paraproteinaemia and a clonal plasma-cell population. The repeated finding of a low plasma-cell burden did not correlate with the extensive skeletal disease on imaging. Targeted biopsy of a lytic lesion ultimately identified a cytokeratin-positive vascular malignancy, leading to the diagnosis of epithelioid angiosarcoma at multidisciplinary review. This case highlights the importance of investigating clinicopathological discordance and avoiding attribution of extensive skeletal disease to an accompanying clonal plasma-cell disorder when the pathological findings do not adequately account for the disease burden. This report was prepared in accordance with the CARE guidelines [5].

## Case presentation

A man in his 80s presented with a three-week history of lower back pain without preceding trauma. His past medical history included a posterior circulation ischaemic stroke, two previous aortic valve replacements, ischaemic heart disease, hypertension, an abdominal aortic aneurysm under surveillance, stage 3 chronic kidney disease, previous cholecystectomy and intermittent self-catheterisation for urinary retention. He was independent in his activities of daily living and had a good functional baseline. On examination, he had no focal upper or lower motor neurone signs and no red flag signs of cauda equina syndrome. Closer assessment of the spine revealed some generalised tenderness but was otherwise normal.

On admission, his haemoglobin was 109 g/L, white cell count was 21.3 × 10⁹/L, C-reactive protein (CRP) was 106 mg/L and adjusted calcium was 2.70 mmol/L (Table 1). Day 1 chest radiography demonstrated left perihilar consolidation (Figure 1), while lumbar spine radiography identified an L3 wedge compression fracture (Figure 2). He was medically managed with intravenous (IV) antibiotics for community-acquired pneumonia and analgesia and conservative management for a presumed osteoporotic vertebral wedge fracture. Mild hypercalcaemia was managed with IV fluids. He was admitted under the geriatric medicine team for inpatient treatment.

**Table 1:** Admission blood tests. Abnormal results are shown in bold. CRP, C-reactive protein

Test parameter	Result	Unit	Reference range
Haemoglobin	109	g/L	130-180
White cell count	21.3	10^9^/L	4.0-11.0
CRP	106	mg/L	<5
Adjusted calcium	2.70	mmol/L	2.20-2.60

**Figure 1:**
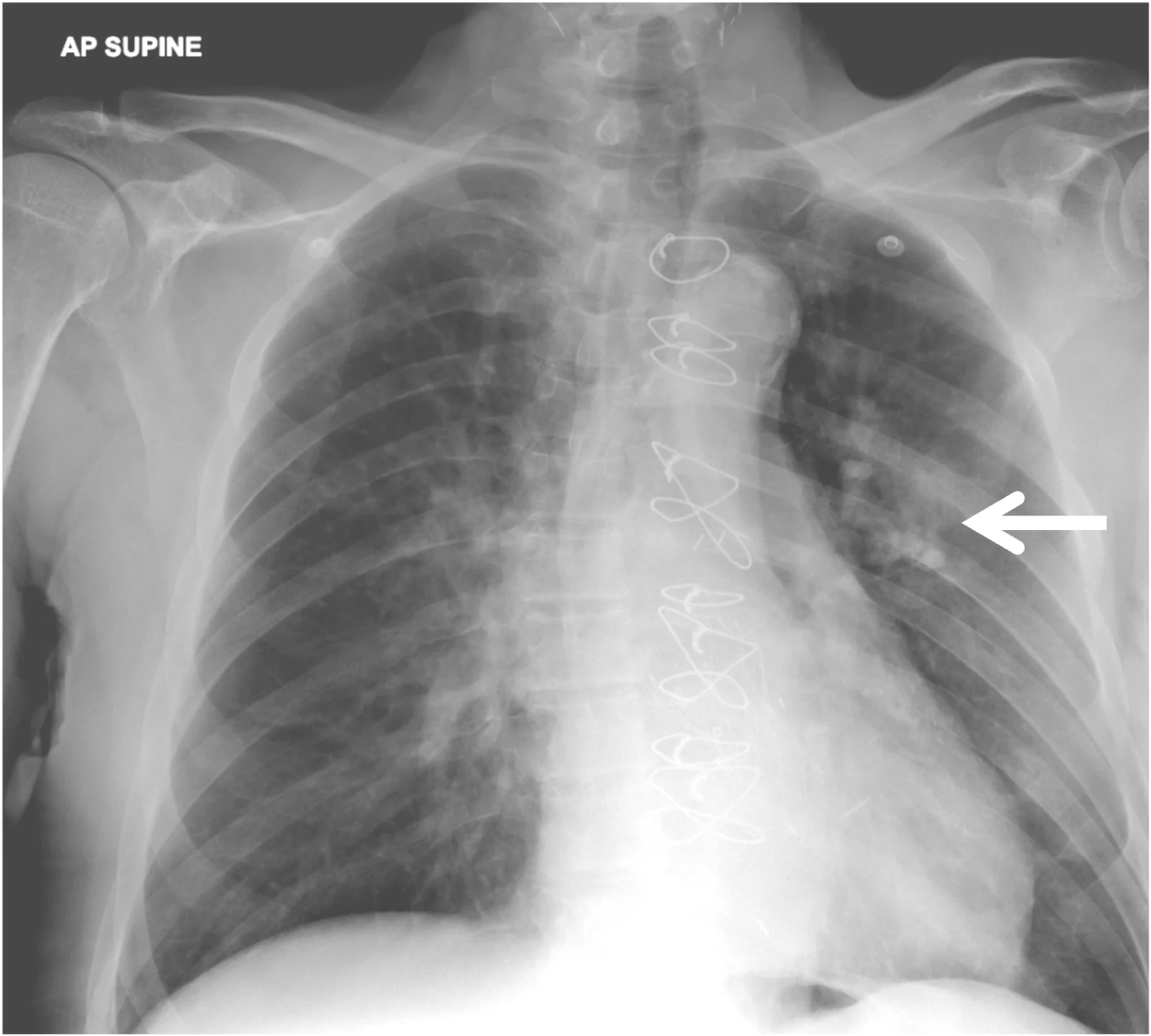
Day 1 chest radiograph showing left perihilar consolidation (white arrow).

**Figure 2:**
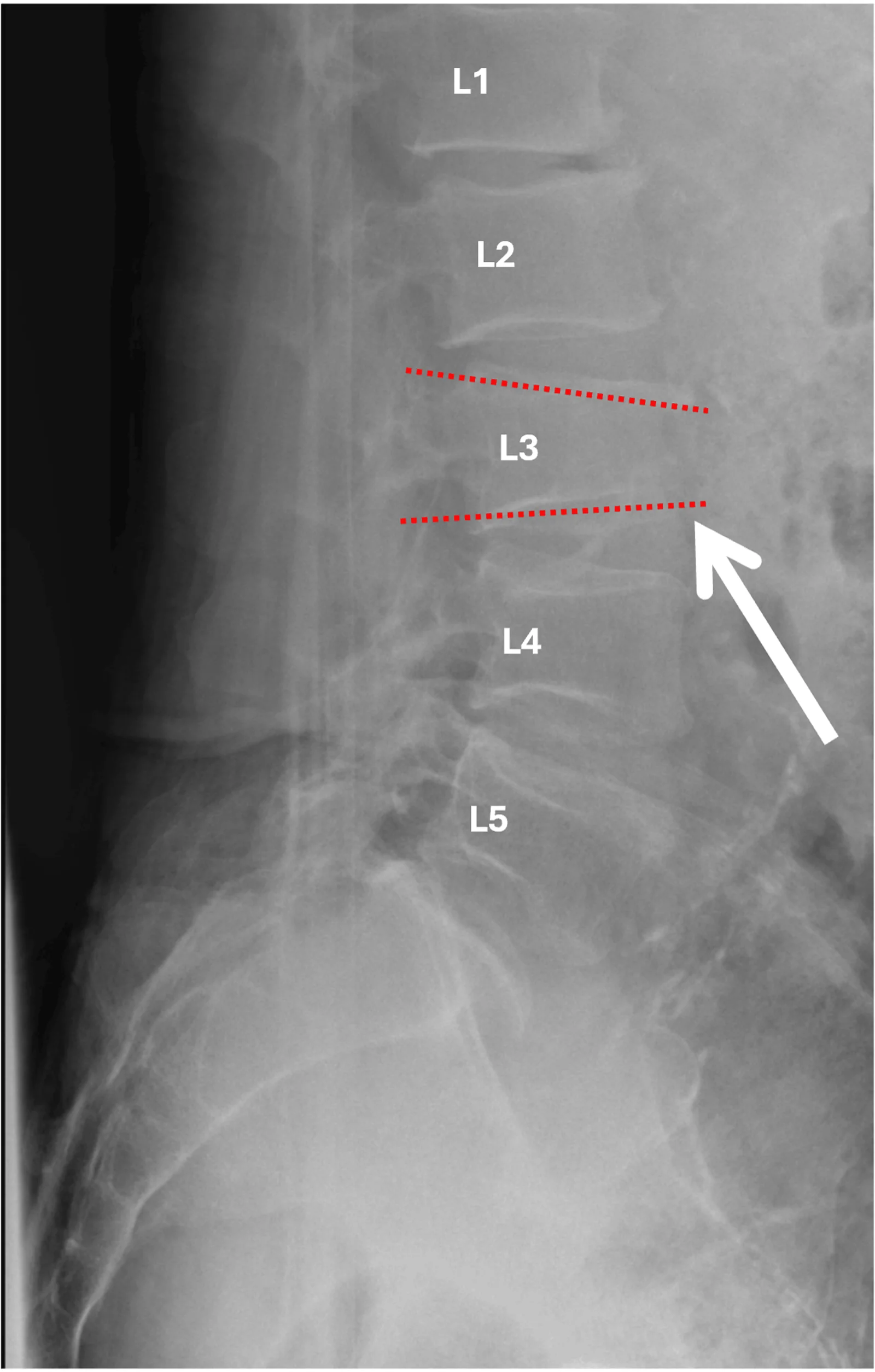
Day 1 lumbar spine radiograph showing an L3 vertebral body wedge compression fracture (white arrow) with anterior loss of vertebral height (red dotted lines).

Due to the findings of hypercalcaemia, back pain and normocytic anaemia, a myeloma screen was sent on Day 2 and showed an abnormal kappa/lambda free light-chain ratio of 6.05. Furthermore, abnormal monoclonal bands were noted on serum electrophoresis. A monoclonal band had also been noted incidentally during serum testing in 2020. Adjusted calcium was once again raised in the Day 2 blood tests and remained between 2.70 and 2.95 mmol/L during the course of the admission (Table 2).

**Table 2:** Serum tests and myeloma screen. Full blood count, urea and electrolytes, C-reactive protein (CRP), bone profile, serum free light chains and serum paraprotein. Abnormal results are shown in bold.

Full blood count	Result	Reference range
Haemoglobin	103	130-180 g/L
White cell count	12.6	4.0-11.0 × 10^9^/L
Platelet count	228	150-400 × 10^9^/L
Haematocrit	0.31	0.40-0.52 L/L
Red cell count	3.47	4.5-6.5 × 10^12^/L
Red cell distribution width	15	11.0%-14.8%
Mean cell volume	88.0	80-100 fL
Mean cell haemoglobin	29.6	27.0-32.0 pg
Neutrophil count	10.66	1.7-7.5 × 10^9^/L
Lymphocyte count	0.83	1.0-4.5 × 10^9^/L
Monocyte count	0.84	0.2-0.8 × 10^9^/L
Eosinophils	0.21	0.0-0.4 × 10^9^/L
Basophils	0.05	0.0-0.1 × 10^9^/L
CRP	166	<5 mg/L
Albumin	27	35-50 g/L
Adjusted calcium	2.73	2.20-2.60 mmol/L
Sodium	141	133-146 mmol/L
Potassium	3.5	3.5-5.3 mmol/L
Urea	7.9	2.5-7.8 mmol/L
Creatinine	91	59-104 μmol/L
Kappa free light chains	117.9	3.3-9.4 mg/L
Lambda free light chains	19.5	5.7-26.3 mg/L
Kappa/Lambda ratio	6.05	0.26-1.65
Serum electrophoresis	Monoclonal bands present	Absent
Paraprotein	7	Absent < 75 years, <10 g/L in > 75 years

Subsequent magnetic resonance imaging (MRI) of the spine on Day 3 demonstrated a pathological fracture of the L3 vertebral body associated with a large T1-weighted hypointense lesion (Figure 3). There was no spinal cord or cauda equina involvement. 

**Figure 3:**
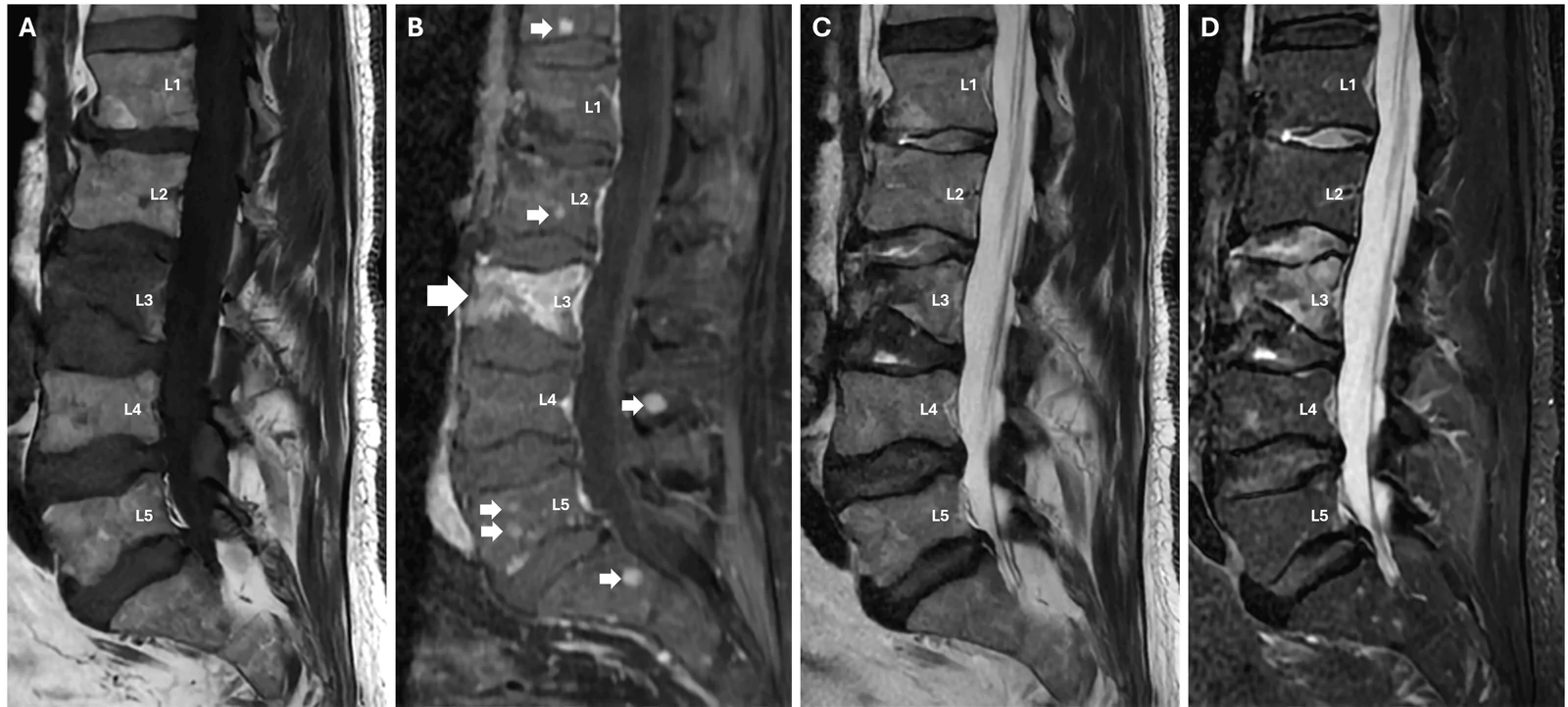
Day 3 MRI of the lumbar spine. (A) Sagittal T1-weighted pre-contrast image showing a low T1 signal lytic lesion within the L3 vertebral body, replacing the normal high T1 signal fatty marrow; (B) sagittal T1-weighted fat-saturated post-contrast image showing enhancement of the L3 vertebral body lesion and multiple enhancing lesions throughout the lumbosacral spine (white arrows); (C) sagittal T2-weighted image showing that the lumbar spinal canal remains capacious, with no cauda equina compromise; and (D) sagittal short tau inversion recovery (STIR) image showing high STIR signal within the L3 vertebral body, consistent with an acute pathological fracture and associated oedema.

Computed tomography (CT) of the chest, abdomen and pelvis demonstrated multiple lytic skeletal lesions but did not identify a primary malignancy (Figure 4). The known abdominal aortic aneurysm was stable compared with previous imaging, and the report recommended interventional radiology/vascular review.

**Figure 4:**
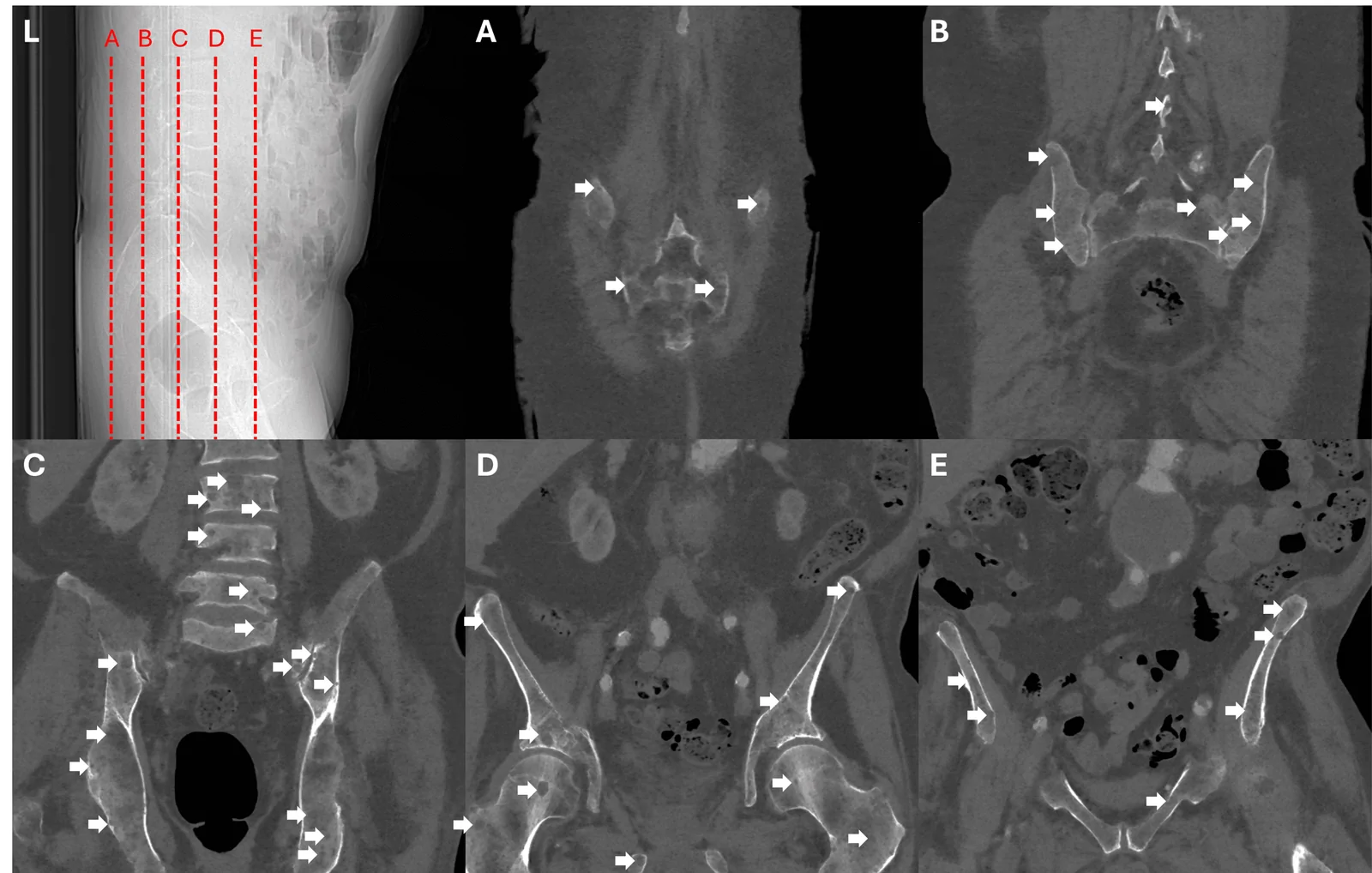
Day 4 CT of the chest, abdomen and pelvis. (L) Sagittal scanogram showing the locations of coronal images A-E; (A-E) coronal minimum intensity projection (MinIP) images demonstrating numerous lytic foci (white block arrows) throughout the lumbar vertebrae, sacrum, bony pelvis and proximal femora.

Clinical timeline 

Day 12 whole-spine MRI identified additional T1-weighted hypointense lytic lesions in the C4, T3, L4, L5 and S1 vertebral bodies (Figure 5). Separately, lesions with signal characteristics of vertebral haemangiomas were identified at C6, T1 and T5 (Figure 5). Without post-mortem correlation, additional foci of vascular tumour at these levels cannot be excluded. The MRI findings were non-specific, with the radiological report commenting that the potential differential diagnoses included metastatic disease and multiple myeloma. 

**Figure 5:**
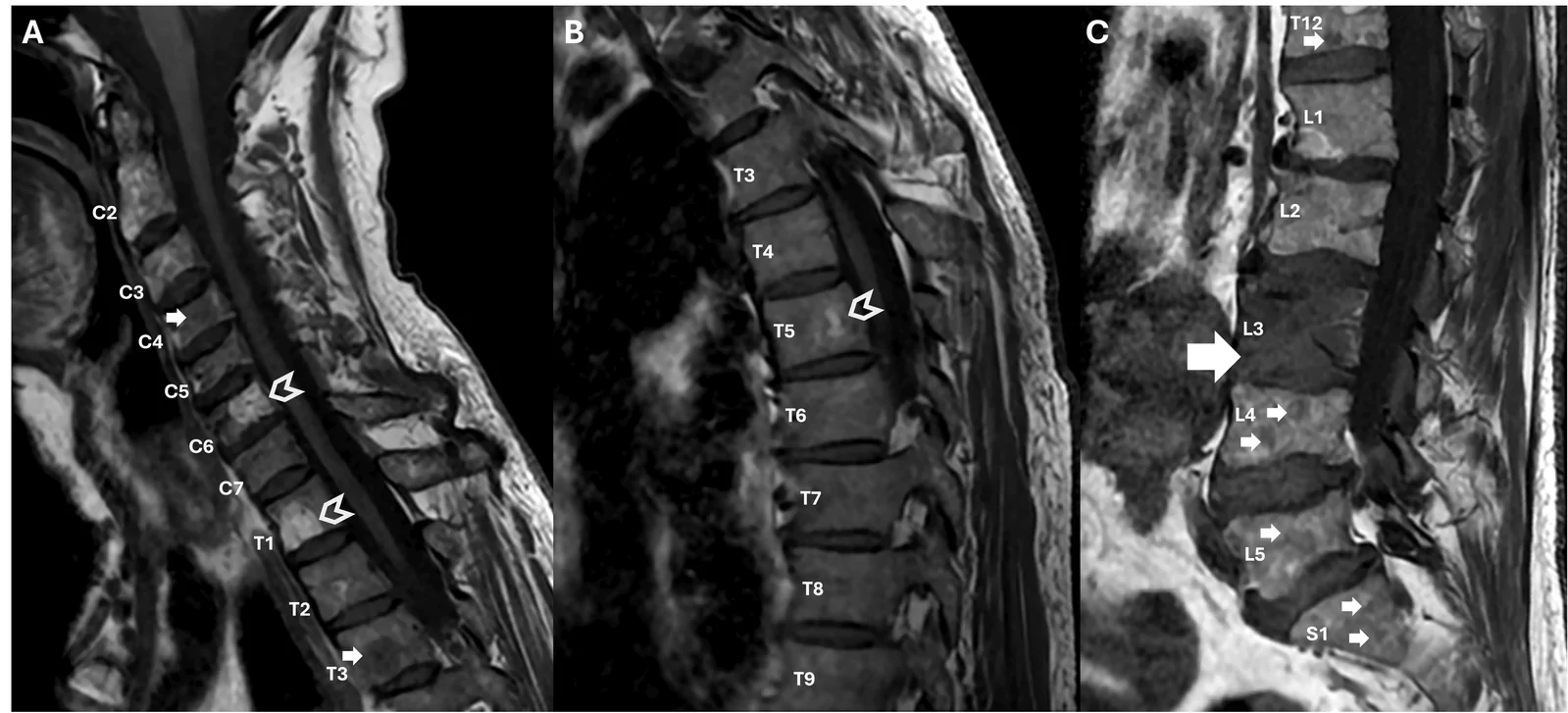
Day 12 sagittal T1-weighted MRI of the whole spine without contrast. (A) Cervical spine; (B) upper thoracic spine; (C) lumbar spine. (A-C) Low-signal lytic lesions (white block arrows) in the C4, T3, L3, L4, L5 and S1 vertebral bodies, replacing the normal high T1 signal fatty marrow, and high-signal lesions (white-bordered chevrons) in the C6, T1 and T5 vertebral bodies, consistent with haemangiomas.

The chronological sequence of events is presented in Table 3.

**Table 3:** Timeline of presentation. Chronological timeline of presentation, key investigations and management, summarising the clinical course. CT, computed tomography; MRI, magnetic resonance imaging

Date	Event
Day 1	Admitted with a three-week history of back pain, raised inflammatory markers, no preceding trauma and no neurological deficit. Chest radiograph demonstrated left perihilar consolidation; lumbar radiograph demonstrated an L3 wedge compression fracture.
Day 2	Myeloma screen demonstrated a kappa/lambda free light-chain ratio of 6.05 and an abnormal monoclonal band on serum electrophoresis. Hypercalcaemia persisted.
Day 3	MRI of the lumbar spine demonstrated multiple small T1-hypointense vertebral and sacral lesions and an L3 vertebral fracture, without discitis or canal stenosis.
Day 4	CT of the chest, abdomen and pelvis demonstrated multiple lytic skeletal lesions with no definite primary malignancy; a myeloma work-up was suggested.
Day 7	Bone marrow aspirate and trephine demonstrated 0.82% kappa-restricted plasma cells by flow cytometry and low-level 3-4% kappa-restricted plasmacytosis on a suboptimal trephine.
Day 12	Whole-spine MRI confirmed a pathological L3 fracture and additional lesions at C4, T3, L4, L5 and the sacrum.
Day 21	Haematology multidisciplinary discussion concluded that the marrow findings did not adequately establish multiple myeloma; targeted biopsy of a lytic lesion was planned.
Day 34	CT-guided biopsy of the L3 lytic lesion was performed.
Day 42	Palliative care review; dexamethasone 6 mg once daily started without clear clinical response. Histology remained pending.
Day 46	Following clinical deterioration and multidisciplinary review, the patient commenced an end-of-life care pathway.
Day 47	The patient passed away.
Day 48	L3 biopsy demonstrated a low-volume plasma-cell neoplasm and a separate atypical epithelioid cell population demonstrating vascular differentiation. Following multidisciplinary review, a likely diagnosis of epithelioid angiosarcoma was made.

Initial bone marrow aspirate and trephine biopsy

Bone marrow aspiration and trephine biopsy were performed on Day 7. Flow cytometry identified a cytoplasmic kappa-restricted plasma-cell population, comprising 0.82% of total nucleated cells. Morphologically, plasma cells accounted for less than 5% of bone marrow cells. The trephine was suboptimal because of fragmentation and crush artefact. CD138 (syndecan-1)-positive plasma cells represented approximately 3%-4% of nucleated cells and were kappa-restricted, CD56 (neural cell adhesion molecule (NCAM))-negative and cyclin D1-positive (Figure 6).

**Figure 6:**
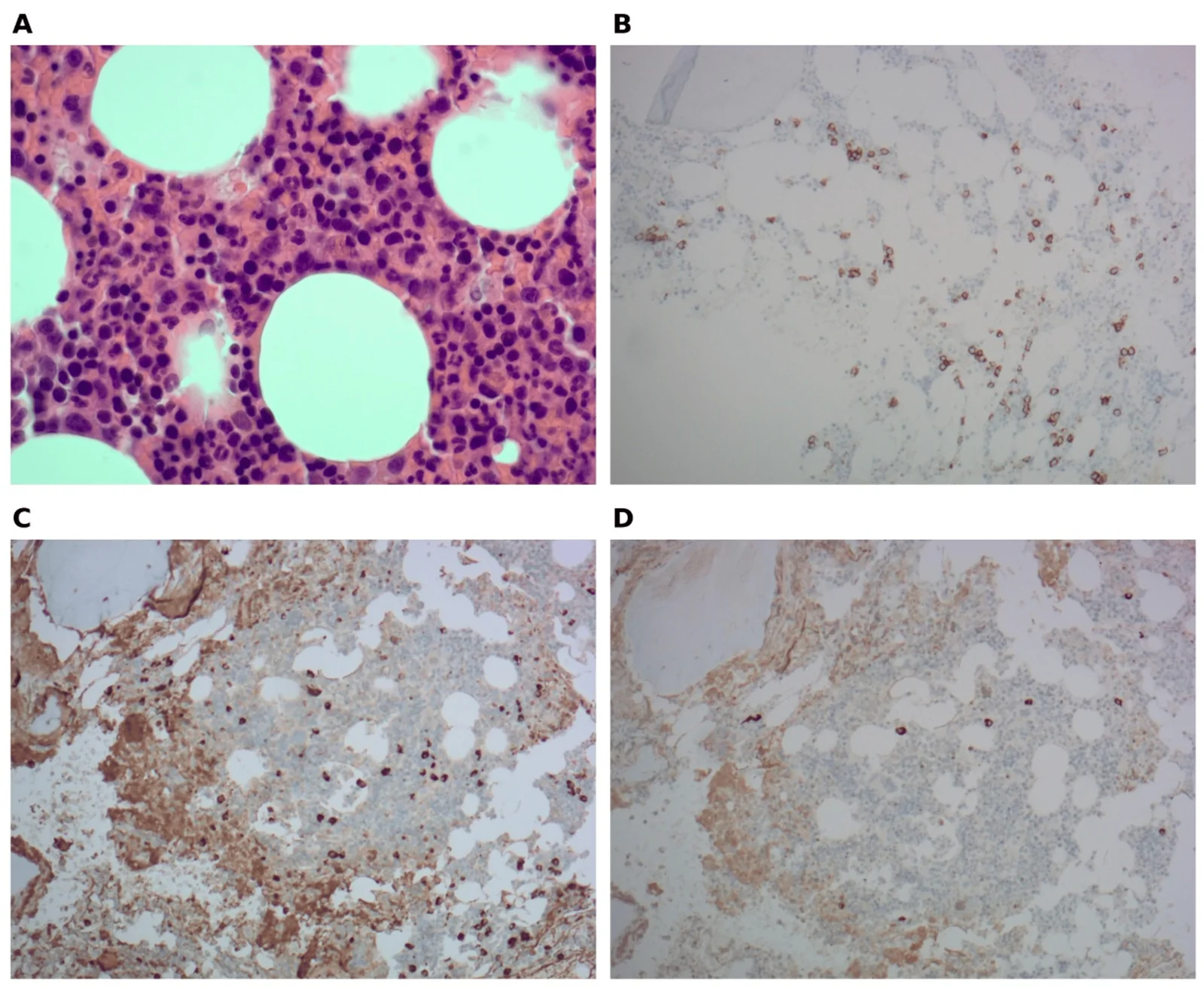
Histological and immunohistochemical findings from the bone marrow trephine, demonstrating a low-level plasma-cell neoplasm. (A) Haematoxylin and eosin (H&E) staining demonstrating bone marrow morphology. (B) CD138 immunohistochemistry highlighting the plasma-cell population. (C) Kappa light-chain immunohistochemistry demonstrating kappa expression within the plasma-cell population. (D) Lambda light-chain immunohistochemistry demonstrating comparatively limited lambda expression, supporting light-chain restriction.

These findings supported a low-level plasma-cell neoplasm. No amyloid deposition was identified. Supplementary immunohistochemical examination of the marrow was also performed and identified two tiny foci of cohesive cells staining positive for pan-cytokeratin (clones AE1/AE3) and low-molecular-weight cytokeratin (clone CAM5.2) (Figure 7).

**Figure 7:**
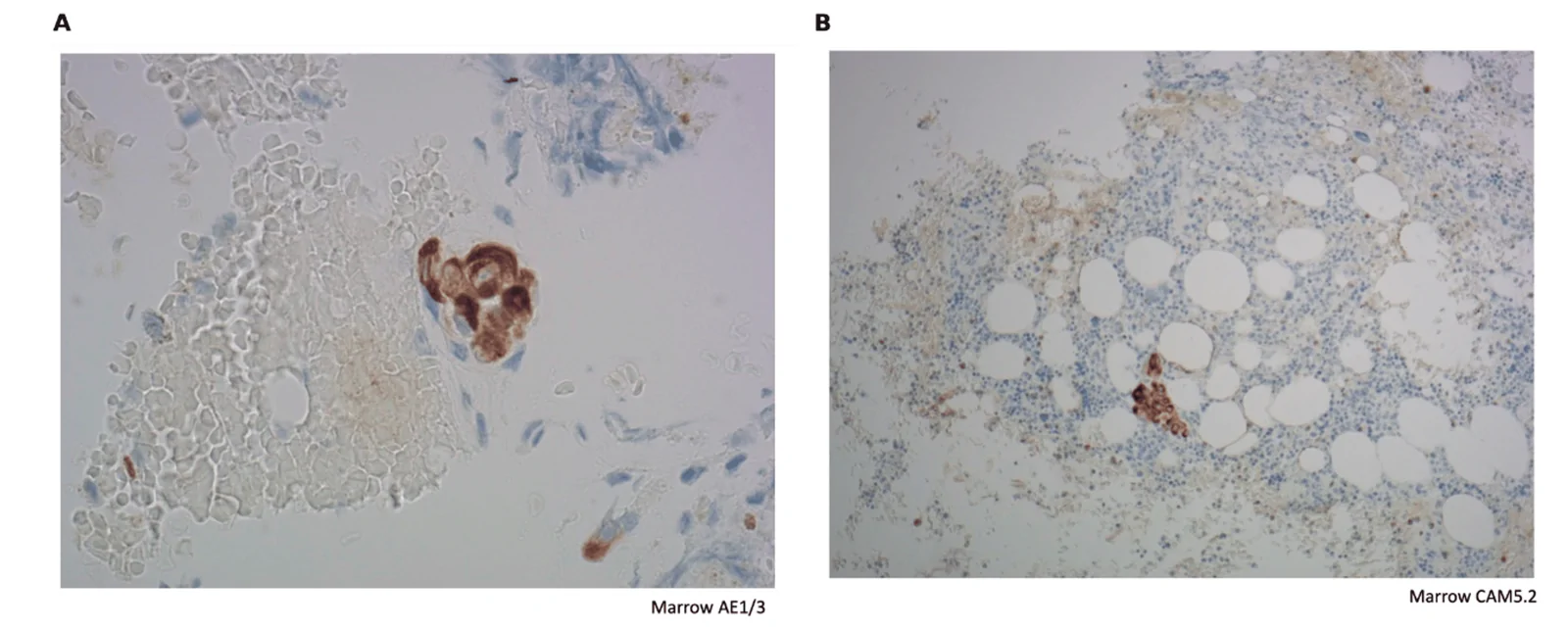
Cytokeratin immunohistochemistry of the bone marrow trephine biopsy. (A) Pan-cytokeratin staining highlighting a small cohesive focus of cytokeratin-positive cells. (B) CAM5.2 staining demonstrating cytokeratin expression in a small cellular focus. These findings raised suspicion of metastatic carcinoma but were not diagnostic of a specific primary malignancy.

These cells were negative for cytokeratin 7 (CK7), cytokeratin 20 (CK20), prostate-specific antigen (PSA), NKX3.1, caudal-type homeobox 2 (CDX2) and thyroid transcription factor 1 (TTF1), initially raising concern for marrow involvement by an occult metastatic carcinoma. Subsequent staining for synaptophysin, chromogranin, tumour protein p63, cytokeratin 5 (CK5) and cytokeratin 6 (CK6) was also negative.

Targeted L3 Bone Biopsy 

As the low plasma-cell burden did not account for the extensive skeletal disease expected in multiple myeloma, a CT-guided biopsy of the L3 lytic lesion was performed on Day 34 (Figure 8).

**Figure 8:**
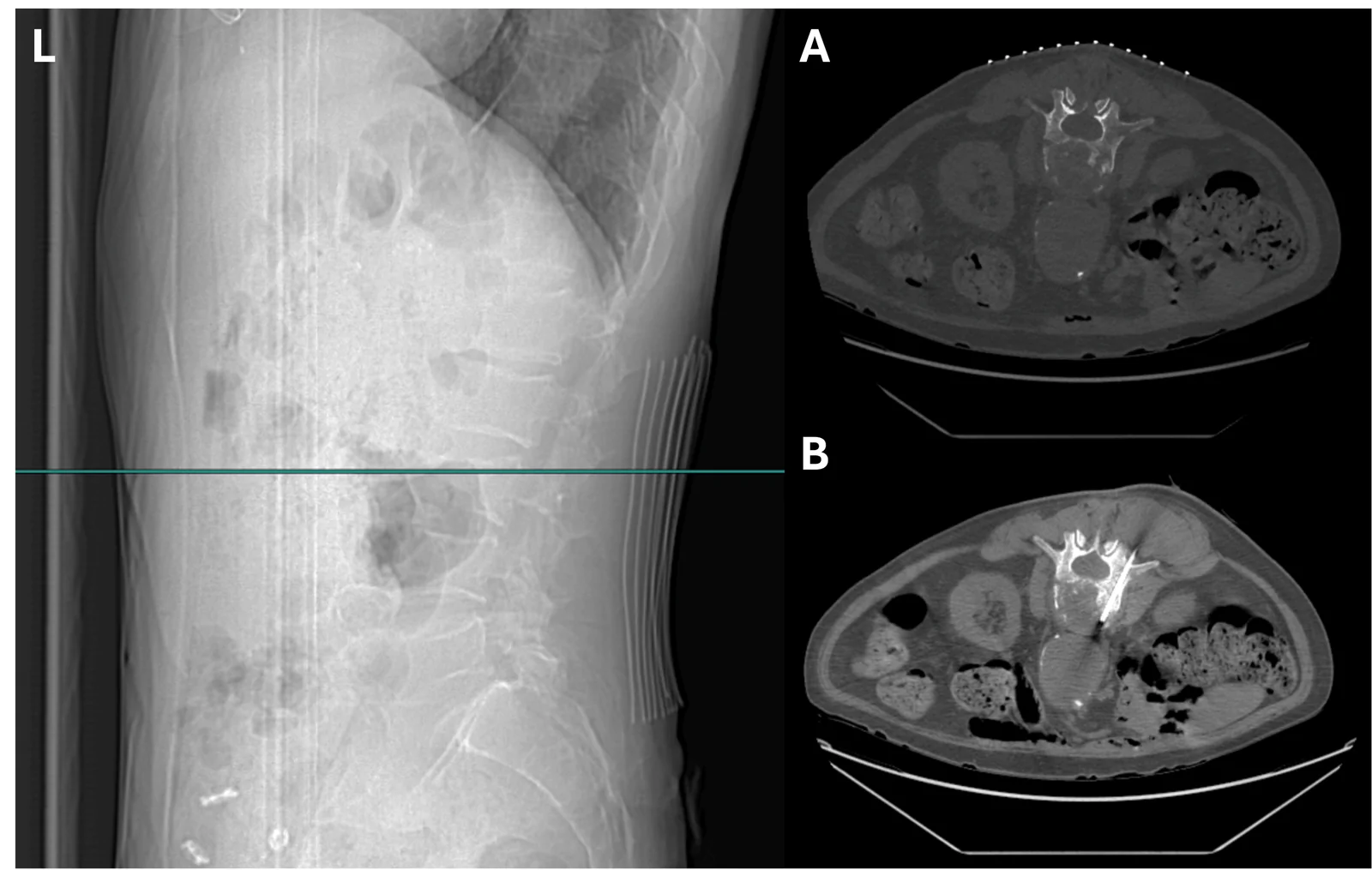
Day 34 CT-guided biopsy of the L3 lesion. (L) Sagittal localiser, with the green line indicating the level of axial image (B); (A) axial slice from the planning volume showing the lytic lesion within the L3 vertebral body; (B) axial slice from the biopsy step-and-shoot showing the biopsy needle within the lytic lesion.

The biopsy mainly consisted of blood with a few fragments of entrapped marrow. Histological examination was performed and again demonstrated a low-volume plasma-cell neoplasm, comprising approximately 4%-5% kappa light-chain-restricted, CD56-negative, cyclin D1-positive plasma cells (Figure 9). 

**Figure 9:**
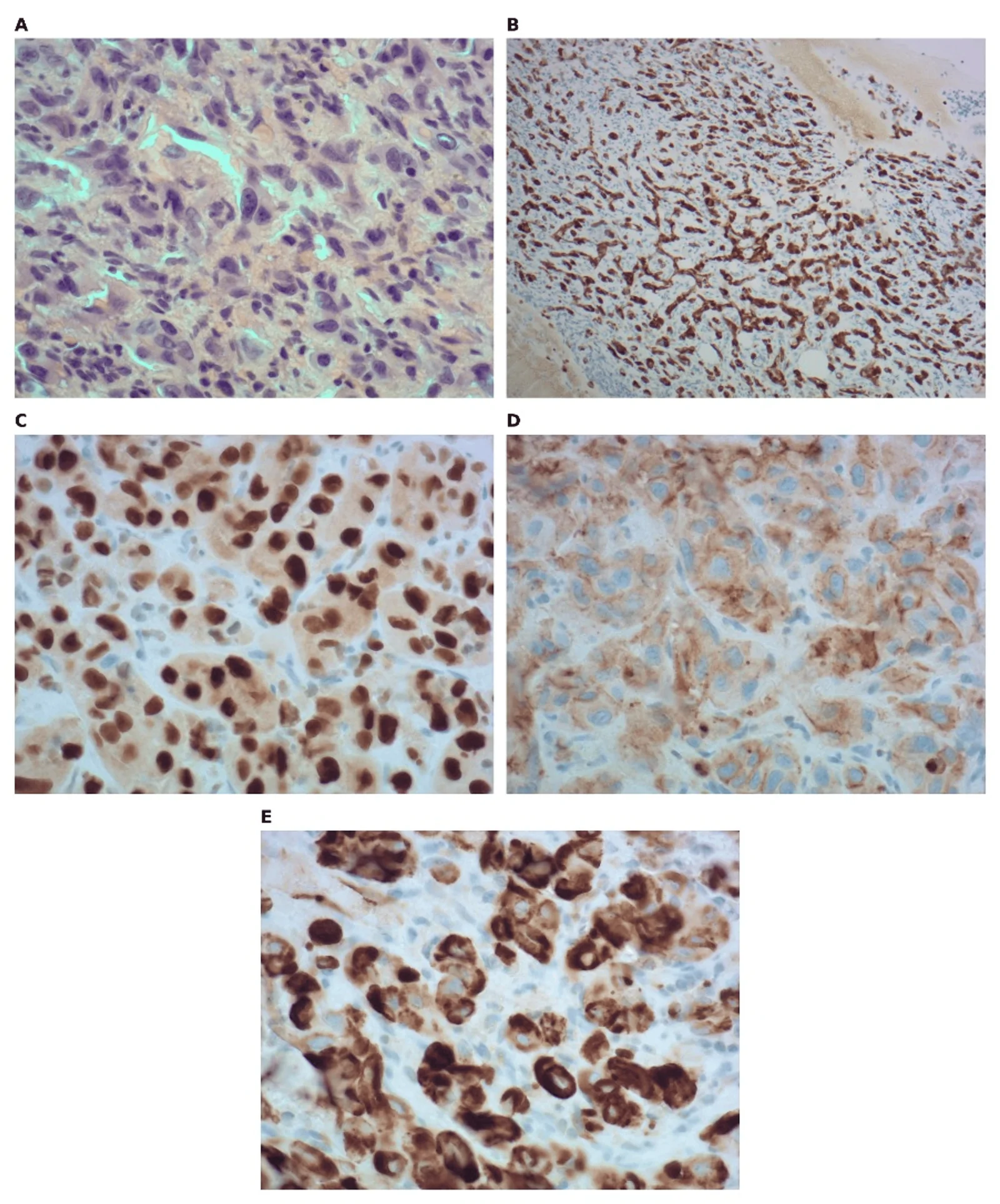
Histopathological and immunohistochemical findings from the L3 bone biopsy. (A) H&E staining demonstrating an atypical epithelioid cell population. (B) Pan-cytokeratin immunohistochemistry demonstrating cytokeratin positivity within the lesional cells. (C) ETS-related gene (ERG) immunohistochemistry demonstrating nuclear positivity within the lesional cells. (D) Platelet endothelial cell adhesion molecule-1 (CD31) immunohistochemistry demonstrating positivity within the lesional cells. (E) Wilms' tumour 1 (WT1) immunohistochemistry demonstrating cytoplasmic positivity within the lesional cells. The combined morphological and immunohistochemical findings support a vascular neoplasm with aberrant cytokeratin expression.

One fragment also contained a separate population of atypical medium-to-large cells, some of which demonstrated epithelioid morphology (Figure 9). Occasional mitoses were seen within these cells, and there was haemosiderin in the background of the fragment. There was no glandular formation and no clear intracytoplasmic vacuoles.

Immunohistochemistry showed that the atypical cells were positive for pan-cytokeratin, ERG, CD31 and cyclin D1, with cytoplasmic Wilms' tumour 1 (WT1) positivity (Figure 9). CD34 and HHV8 were negative in the lesional cells, with CD34 highlighting background vessels only (Table 4). A broad additional panel was negative for CK5/6, CK7, CK19, CK20, MOC31, BerEP4, TTF1, NKX3.1, PAX8, GATA3, CDX2, CD30, hepatocyte-specific antigen (HSA), chromogranin, synaptophysin, calretinin and SOX10 (Table 4).

**Table 4:** Immunohistochemical profile of the atypical epithelioid cell population identified in the L3 bone biopsy. CD34, cluster of differentiation 34; TTF1, thyroid transcription factor 1; HSA, hepatocyte-specific antigen

Marker	Result in atypical lesional cells	Interpretation
Pan-cytokeratin (clones AE1/AE3)	Positive	Demonstrates epithelial/cytokeratin expression; aberrant expression can occur in epithelioid vascular neoplasms
ETS-related gene (ERG)	Positive	Supports endothelial differentiation
Platelet endothelial cell adhesion molecule-1 (CD31)	Positive	Supports endothelial differentiation
Wilms' tumour 1 (WT1)	Cytoplasmic positivity	Non-specific cytoplasmic staining; interpreted in conjunction with the overall immunophenotype
Cyclin D1	Positive	Non-specific; expressed in many carcinomas and sarcomas, including some vascular tumours
CD34	Negative in lesional cells; background vessels highlighted	Does not exclude vascular differentiation; provides an internal positive control in background vessels
Human herpesvirus 8 (HHV8)	Negative	Does not support HHV8-associated Kaposi sarcoma
Cytokeratin (CK5/6, CK7, CK19, CK20)	Negative	Does not support the corresponding epithelial immunophenotypes
MOC31, BerEP4	Negative	Lack of epithelial marker expression argues against conventional metastatic carcinoma in the appropriate context
TTF1, NKX3.1, PAX8, GATA3, CDX2	Negative	No immunophenotypic support for common lung, prostate, renal/Müllerian, breast/urothelial or gastrointestinal primary sites
CD30, HSA, chromogranin, synaptophysin, calretinin, SOX10	Negative	No support from these markers for the corresponding alternative lineages/differential diagnoses

The combination of epithelioid morphology, endothelial marker expression and aberrant cytokeratin expression indicated a separate vascular malignancy, rather than part of the plasma-cell process. Following a multidisciplinary review of the clinical, radiological, biochemical and histopathological findings, epithelioid angiosarcoma was agreed to be the likely diagnosis.

Diagnosis

Differential Diagnosis

Given the multifocal lytic bone lesions, persistent hypercalcaemia, abnormal kappa/lambda ratio and presence of monoclonal bands on serum electrophoresis, multiple myeloma was strongly suspected initially. It was a particularly compelling differential diagnosis due to the presence of kappa-restricted plasma cells on flow cytometry. Metastatic carcinoma from an occult primary was also considered, particularly after the initial bone marrow examination demonstrated tiny cytokeratin-positive cohesive foci. However, CT of the chest, abdomen and pelvis did not identify a primary malignancy, and subsequent immunohistochemistry did not support an epithelial primary.

*Outcome* 

Histological examination of the targeted L3 biopsy on Day 34 demonstrated immunophenotypic markers and cell morphology correlating with a separate, distinct epithelioid malignancy. Importantly, it created a diagnostic split comprising two pathological components: a low-volume kappa-restricted plasma-cell neoplasm, accounting for approximately 3%-4% of the trephine biopsy and 4%-5% of the lesional biopsy, respectively; and a separate population of atypical epithelioid cells expressing endothelial markers ERG and CD31, together with pancytokeratin AE1/AE3.

By the time the diagnostic picture had become clearer, the patient's clinical condition had deteriorated significantly. Hypercalcaemia persisted despite treatment, and a trial of dexamethasone for malignancy-associated back pain did not result in symptomatic improvement. Following multidisciplinary and palliative care review on Day 42, the patient was initiated on the end-of-life pathway on Day 46 and passed away the next day. On Day 48, following multidisciplinary correlation of the histological, immunophenotypic, radiological and clinical findings, the final MDT outcome was epithelioid angiosarcoma coexisting with a low-volume plasma-cell neoplasm.

## Discussion

This case illustrates an unusual diagnostic puzzle as a result of two pathological abnormalities. The ongoing low plasma-cell burden was the key conflicting feature in determining the underlying diagnosis. Although a clonal plasma-cell population was present, its limited extent did not provide a satisfactory pathological explanation for the extensive spinal lesions. The patient had biochemical and pathological evidence of a low-volume clonal plasma-cell disorder while simultaneously harbouring immunophenotypic features of an epithelioid vascular neoplasm with bone involvement. In the setting of multifocal osteolytic lesions and hypercalcaemia, the additional presence of a monoclonal paraprotein and kappa-restricted plasma cells understandably favoured multiple myeloma as the initial diagnosis.

This distinction is clinically important because identification of a monoclonal gammopathy does not necessarily establish that a plasma-cell clone is responsible for bone lesions. The International Myeloma Working Group criteria require ≥10% clonal plasma cells or a biopsy-proven bony/extramedullary plasmacytoma, plus a myeloma-defining event, to qualify for a formal diagnosis of multiple myeloma [1]. In this patient, clonal plasma cells accounted for 3%-5% in both the marrow and the lesional biopsy, and the targeted lytic lesion did not contain a plasmacytoma. Hence, in this case, a low-level clonal plasma-cell population requires interpretation in the wider clinical and pathological context. Once the bone disease and hypercalcaemia were attributed to angiosarcoma, the plasma-cell findings were retrospectively most consistent with a monoclonal gammopathy of undetermined significance (MGUS), present since at least 2020; criteria for smouldering myeloma were not met (clonal plasma cells <10%, paraprotein 7 g/L), although formal classification was limited by the short clinical course. Flow cytometric quantification may also underestimate plasma-cell burden, particularly where marrow involvement is focal or the aspirate is suboptimal, reinforcing the need to correlate aspirate findings with trephine morphology [6].

Although a pathogenetic link between MGUS and epithelioid angiosarcoma cannot be entirely excluded, the two processes were histologically distinct. This case is therefore best regarded as an incidental collision of a long-standing, low-volume monoclonal gammopathy, which is common in older adults [7], with a rapidly progressive vascular malignancy, which contributed to the initial anchoring bias.

Angiosarcoma of the bone is rare, accounting for less than 1% of malignant bone tumours [2]. Published cases demonstrate that osseous angiosarcoma can present with multifocal osteolytic disease closely resembling multiple myeloma [2,3]. A recent report of metastatic multicentric epithelioid angiosarcoma of bone similarly described hypercalcaemia and highlighted the diagnostic pitfalls associated with this rare malignancy [8]. The present case is additionally complicated by the coexistence of a clonal plasma-cell neoplasm, which strengthened the initial clinical impression of multiple myeloma.

Epithelioid angiosarcoma can mimic metastatic carcinoma. Cytokeratin expression is most commonly associated with carcinoma but, due to endothelial differentiation, can also be positive in epithelioid angiosarcoma, creating diagnostic challenges. In a series of seven epithelioid angiosarcomas involving bone and soft tissue, all cases expressed CD31 and six of seven expressed cytokeratin; initial biopsies in some patients were interpreted as suggestive of metastatic carcinoma [4]. This closely parallels the diagnostic evolution in the present case. Tiny AE1/3- and CAM5.2-positive foci in the original marrow initially raised concern for occult metastatic carcinoma. Targeted lesional biopsy subsequently demonstrated an epithelioid population expressing ERG and CD31, establishing vascular differentiation and ultimately supporting the diagnosis of epithelioid angiosarcoma.

The diagnostic cornerstone of this case was the marked discordance between the patient’s substantial radiological tumour burden and the unexpectedly low plasma-cell infiltration observed on initial bone marrow sampling. As plasma-cell dyscrasias and architecturally dependent marrow lesions frequently present with focal, patchy involvement, routine bone marrow aspirate and trephine biopsies can yield discordant or false-negative results [9]. Performing a radiologically guided biopsy was essential in preventing the incorrect attribution of osteolytic lesions to a low-grade plasma-cell disorder and successfully unmasking the secondary, coexisting pathological process.

The case also demonstrates the potential dangers of diagnostic anchoring. The following findings, namely extensive lytic lesions, hypercalcaemia, monoclonal gammopathy and a kappa-restricted plasma-cell population, collectively supported the initial hypothesis of multiple myeloma. Yet, when considered alongside the unexpectedly low plasma-cell burden, the findings did not provide a coherent explanation for the extent of the patient's disease. Recognition of this discrepancy prompted targeted biopsy and ultimately revealed epithelioid angiosarcoma.

Significantly, the diagnostic challenge extended beyond differentiating epithelioid angiosarcoma from multiple myeloma. Cytokeratin expression initially suggested an epithelial malignancy, demonstrating how epithelioid angiosarcoma may imitate both a haematological malignancy clinically and metastatic carcinoma histologically. Therefore, identification of endothelial differentiation through ERG and CD31 expression, combined with clinico-radiological correlation and multidisciplinary review, was central to establishing the final diagnosis.

This report has some limitations. PET-CT and post-mortem examination were not performed, so we could not fully assess the extent of disease or rule out a primary tumour outside the bone. As a result, there remains some level of diagnostic uncertainty as to whether the bone lesions represented multicentric primary angiosarcoma of bone or metastases from an undetected primary. In addition, the lesional tissue was largely consumed by immunohistochemical testing, which limited further investigations. Finally, 13 days passed between the decision to biopsy (Day 21) and the biopsy itself (Day 34), and the diagnosis was only reached after the patient had deteriorated significantly. In retrospect, the discordance between imaging and the Day 7 marrow findings could itself have justified earlier targeted lesional biopsy, rather than awaiting MDT consensus.

## Conclusions

Epithelioid angiosarcoma of bone is a rare malignancy that can present with multifocal lytic lesions and mimic multiple myeloma. This case was particularly challenging because a low-volume plasma-cell population, monoclonal paraprotein and hypercalcaemia created a highly plausible alternative explanation for the extensive skeletal disease. 

The discrepancy between the limited plasma-cell burden and the extensive radiological abnormalities was the crucial clue that prompted targeted lesional biopsy. The subsequent identification of an epithelioid malignancy expressing both endothelial markers and cytokeratin ultimately led to the diagnosis of epithelioid angiosarcoma. 

This case highlights two important diagnostic pitfalls: the risk of attributing extensive lytic disease to an incidental or low-volume plasma-cell clone, and the potential for cytokeratin-positive epithelioid angiosarcoma to be mistaken for metastatic carcinoma. When histopathological findings do not match the clinical and radiological picture, the working diagnosis should be reconsidered and targeted biopsy of radiologically abnormal tissue pursued. 
